# Differences in the Racial Contribution of Dementia and Chronic Conditions to Hospitalization, SNF Admission

**DOI:** 10.1093/geroni/igab046.1066

**Published:** 2021-12-17

**Authors:** Heather Allore

**Affiliations:** Yale School of Medicine, New Haven, Connecticut, United States

## Abstract

We estimate the contribution for experiencing hospitalization, skilled nursing facility admission and mortality using a measure of attributable fraction that incorporates both the prevalence, incidence and risk called Longitudinal Extension of the Average Attributable Fraction (LE-AAF). We estimate the LE-AAF for Non-Hispanic whites and Non-Hispanic Blacks for dementia and 10 chronic conditions, for three outcomes. This approach analyses the temporal relationships among conditions to estimate their population-level average attributable fractions. Unlike standard measures of attributable fraction, the sum of the contribution of each condition based on the LE-AAF will not exceed 100 percent, enabling us to compute the contribution of pairs, triads or any combination of conditions. Furthermore, in studying multimorbidity, the LE-AAF has the desirable feature of being based on all combinations of the risk factors and covariates present in the data with final values for the individual LE-AAFs obtained by averaging across these observed combinations of predictors.

